# Evidence for Mitotic Recombination within the Basidia of a Hybrid Cross of *Cryptococcus neoformans*

**DOI:** 10.1371/journal.pone.0062790

**Published:** 2013-05-14

**Authors:** Aaron A. Vogan, Jordan Khankhet, Jianping Xu

**Affiliations:** Department of Biology, McMaster University, Hamilton, Ontario, Canada; College of Forestry, Central South University of Forestry and Technology, Changsha, Hunan Province, China; University of Minnesota, United States of America

## Abstract

In the majority of diploid eukaryotes, each meiotic process generates four haploid gametes with each containing a single recombinant nucleus. In some species and/or some meiotic processes, aneuploid or diploid gametes can also be generated due to chromosomal non-disjunction and/or the co-packaging of two of the four haploid nuclei into the same gamete. Here we show that another process is involved in generating genotypes of sexual progeny from a hybrid cross between two divergent lineages of the human fungal pathogen *Cryptococcus neoformans*. Through micro-dissection of 1358 basidiospores from 194 basidia and genotyping using 33 co-dominant genetic markers, the genotypes of all 230 germinated basidiospores from 94 basidia were obtained. The minimum haploid genotypes required to constitute the observed genotypes from each basidium were then inferred. Our results demonstrated that more than four haploid nuclear genotypes are required to explain the observed genotypes of basidiospores in seven of the 94 basidia. Our results suggest that mitotic recombination within basidia must be involved to produce the observed genotypes in these seven basidia. The mitotic recombination likely includes both chromosomal loss and crossing over. This novel recombination process could play an important role in generating the genotypic and phenotypic diversities of this important human pathogen.

## Introduction

Since the first discovery of meiosis in the late 1800’s in sea urchin eggs [1], the fundamental genetic features of meiosis have proven to be virtually universal for sexual reproduction throughout the Eukarya Domain [2]. Specifically, a typical meiosis involves one round of genome duplication, followed by pairing and crossing-over between homologous chromosomes and two rounds of reductive division to generate four genetically different nuclei with each having half of the nuclear genetic material as the original cell. Some of the cytological processes in meiosis vary among organisms. For example, in the fungal subkingdom Dikarya, which includes mushrooms, yeasts, and molds, sexual reproduction includes the dikaryotic phase – a binucleate hyphal structure formed from the fusion of two haploid monokaryotic individuals with compatible mating types. In most cases, the haploid nuclei remain separate in the dikaryon and only fuse to form a single diploid nucleus right before meiosis. The diploid nucleus then undergoes one replication and two reductive divisions to produce four haploid recombinant daughter nuclei, which are subsequently packaged into four sexual spores [3], [4].

*Cryptococcus neoformans* is a dimorphic basidiomyceteous fungus, consisting of a haploid, asexual yeast form and a dikaryotic, sexual filamentous form [5]. It is an opportunistic human pathogen, infecting up to one million people a year [6]. Its medical significance and ease of genetic manipulation in the laboratory have made *C. neoformans* a model organism for fungal pathogen research [7], [8]. *C. neoformans* is composed of two varieties var. *grubii* and var. *neoformans*, which have been traditionally identified as serotype A and serotype D respectively based on their cell surface antigenic properties. Different from sexual reproduction in the majority of basidiomycetes, the production of four haploid daughter nuclei in each *C. neoformans* basidium is typically followed by multiple rounds of mitosis, with each haploid nucleus entering into one spore and each basidium bearing four chains of basidiospores. Interestingly, basidiospores from each of the four chains are genetically heterogeneous, suggesting that haploid nuclei in each basidium are randomly distributed into the spores [9], [10]. Analyses of micro-dissected chains of basidiospores from intra-variety crosses (i.e. between serotype A strains and between serotype D strains) have revealed that only four haploid genotypes are found for spores isolated from each basidium, consistent with the hypothesis that only one round of meiosis occurs in each basidium [9], [10].

Epidemiological surveys have identified that strains of serotype AD are commonly found in both environmental and clinical populations of *C. neoformans* [11], [12]. In certain geographic regions, serotype AD strains can account for a significant percentage of clinical isolates [12]. Gene genealogical analyses have indicated that serotype AD strains are the results of recent hybridizations between strains of serotypes A and D [13], [14]. Interestingly, in contrast to the haploid status for strains of serotypes A and D, most serotype AD strains are diploid or aneuploid and are heterozygous for at least some loci. These results suggested that sexual reproduction (i.e. meiosis and sporulation) in AD hybrids might be different from that in intra-varietal crosses between serotype A strains or between serotype D strains.

In the genetic analyses of a hybrid cross between strains JEC20 (serotype D, mating type a or MAT**a**) and CDC15 (serotype A, MAT**α**), 157 of the 163 analyzed progeny were found to contain at least one heterozygous locus out of the 114 screened co-dominant loci [15]. The mean heterozygosity per locus was estimated at 74.81% among the 163 progeny. The high level heterozygosity was attributed to non-disjunction during meiosis [15]. However, due to the random nature of the examined progeny population, other potential processes that might have contributed to the observed genotype diversity in the progeny could not be excluded.

The objective of this study is to analyze the processes that might be involved in generating progeny genotypes in a hybrid cross of serotypes A and D. To accomplish this, we dissected 1358 basidiospores from 194 basidia using a micromanipulator. The genotypes of the germinated spores were analyzed using 33 co-dominant genetic markers. Based on the observed genotypes from the germinated spores of each basidium, we infer the minimum number of haploid genotypes and the events that would be required in order to reconstitute the observed genotypes. Our analyses provided evidence for mitotic recombination within the basidia in the inter-variety hybrid cross. Furthermore, the results indicated that the mitotic recombination within the basidia likely included both chromosomal loss and crossing over.

## Materials and Methods

### Hybrid Progeny

Parental strains JEC20 (Serotype D, MATa) and CDC15 (Serotype A, MATα) were mated on V8 agar, following the same protocol as that described in Sun *et al.* (2007) [15]. After 1–4 weeks of incubation at 23°C, basidiospores were collected through microdissection from individual basidia. Specifically, each entire mating spot containing hyphae and basidiospores was first cut from the V8- mating medium and transferred to a slightly bigger hole in a new plate containing the yeast extract - peptone - dextrose (YEPD) medium. Chains of basidiospores from each individual basidium that were well - separated from other chains of basidiospores on other basidia were transferred to separately marked fresh spots on the YEPD medium using a micromanipulator (MSM System 300, Singer Instruments). Individual basidiospores were then picked and transferred to pre-determined spots on the agar to allow easy tracking of the relationships among basidiospores with respect to the dissected basidia. Basidiospores were incubated at 23°C for up to 3 weeks to ensure that slow-germinating and/or slow-growing basidiospores could form colonies for genotyping. DNA was extracted from these colonies using the method described in Xu *et al.* (2000) [16].

### Genotyping

**Figure 1:**
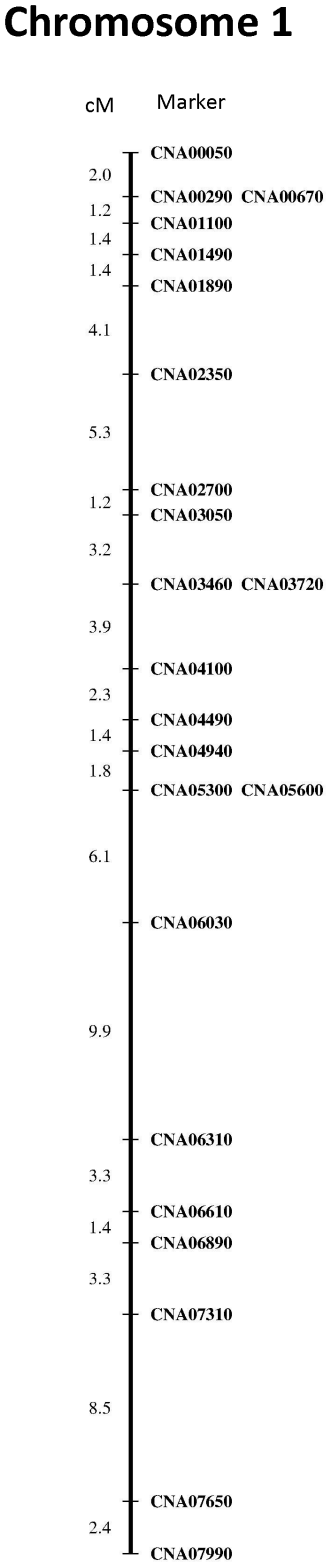
Linkage relationships among the examined PCR-RFLP markers on Chromosome 1. Distances are in centi-Morgan, markers are evenly spaced with ∼1/100 kb. Markers on the same line had less than 1 cM distance between them. No recombination was observed between markers CNA05300 and CNA05600.

A total of thirty-three co-dominant markers were used to genotype all progeny. These include 32 PCR-RFLP markers distributed on 4 chromosomes with 23 markers on Chromosome 1, 4 on Chromosome 3, 2 on Chromosome 4, and 3 on Chromosome 7. The reasons for including a large number of markers for Chromosome 1 were to: (i) help identify potentially multiple recombination breakpoints within individual basidia on one chromosome; (ii) reveal reciprocity of recombinant products at a fine scale; and (iii) investigate potential discordance between the physical map and the linkage map for the largest chromosome in the hybrid cross. Protocols for obtaining the PCR-RFLP genotypes followed that described in Sun *et al*. (2007) [15]. 15 of the 32 markers were the same as those used by Sun et al. (2007) [15] while the remaining 17 markers were designed using Prifi [17] based on the whole genome sequences of JEC21 and H99 (Table S1). In addition to the 32 PCR-RFLP markers, we also screened the mating types of these progeny using both the MAT**a** and MAT**α** –specific primer pairs located on Chromosome 4, following the protocol described in Yan et al. (2003) [18]. The physical relationships among these markers in the JEC21 genome are shown in Figure 1. MapMaker 3.0 was used to construct a linkage map for Chromosome 1. Individuals that showed an identical genotype to another basidiospore from the same basidia were excluded from the mapping population.

### Haplotype Inference

To infer the number of unique haploid genotypes from the observed genotypes for each basidium, we employed two different approaches. In the first, we assumed that any progeny with at least one heterozygous locus were a diploid and all loci containing allele from only one parent were treated as homozygous at these loci (i.e. each with two identical alleles). The observed genotypes from each basidium were then analyzed with the PHASE 2.1 program [19.20] to infer the minimum number of haploid genotypes that were required to explain the observed genotypes of each basidium. In the second approach, we assumed that only heterozygous loci had two alleles and the loci with allele from only one parent are hemizygous, containing only one allele at the specified locus. These genotypes were then used to infer the minimum set of haploid genotypes for each basidium. While the first approach would result in inferred haploid genotypes each with a whole complement of all chromosomes, the second approach would result in certain haploid genotypes with only a subset of the chromosomes.

### Microscopy

To investigate whether individual basidiospores are uninucleated (monokaryotic) or binucleated (dikaryotic), basidiospores were stained and examined using fluorescent microscopy. Briefly, hyphae produced from mating spots (described above) were scraped with a loop and fixed to a slide. The slide was placed in a solution with DAPI and Calcofluor as described by Wickes *et al.* (1996) [21] for 30 minutes to stain the nuclei and cell walls respectively of the basidia and basidiospores for visualization.

## Results and Discussion

1358 basidiospores were individually picked using a micromanipulator and incubated on YEPD agar for up to three weeks. Each progeny that germinated and formed a colony on the agar medium was genotyped using the 33 co-dominant molecular markers. Below we describe the germination rates and genotypes of the progeny, followed by comparisons of the genotype data with those derived from two models that are known to generate atypical meiotic products.

### Basidiospore Germination

Of the 1358 basidiospores that were micro-dissected from 194 basidia, 230 basidiospores (17%) from 94 basidia germinated and grew into full colonies that could be genotyped and analyzed. Our overall germination rate was higher than the 5.5% observed by Lengeler *et al.* (2001) [22] in their meiotic progeny of a serotype AD strain. One factor that could have contributed to the difference in germination rates observed between our study and that by Lengeler *et al.* (2001) [22] was the different parental strains used. Specifically, the parental strain that Lengeler *et al.* (2001) [22] used was a self-fertile natural AD hybrid while ours were two self-sterile strains with different mating types. Their natural hybrid parent strain might be hemizygous for certain loci, lacking certain genetic complements from either the serotype A or D parental strains that could have contributed to a higher percentage of inviable basidiospores. In contrast, our zygotes would contain the whole complement of genetic materials from both the serotypes A (strain CDC15) and D (strain JEC20) parents.

Although a single basidium can potentially produce over 100 basidiospores [9], only 7 basidiospores were dissected for the majority of the basidia examined (159) (Table S2 shows the full distribution of the number of spores plated per basidium). This was done in an effort to obtain a genotypically diverse set of progeny from many basidia. Of the 94 basidia that contained germinated basidiospores, the number of basidiospores germinated ranged from 1 to 7 per basidium, with the mean and median of 2.4 and 2 respectively. Large differences in basidiospore germination rates were found among the dissected basidia. However, due to the relatively low number of basidiospores dissected from each basidium, germination rate comparisons among these basidia were not conducted.

### Distributions of Observed Genotypes and Inferred Haplotypes

All 230 germinated basidiospores were genotyped at the 33 loci (Table S3). The combined multilocus genotype data were used to determine the number of unique genotypes for each basidium. Our analyses identified that the largest number of unique genotypes from one basidium was 6 (Table 1), present in basidium 51 (Table S3). For the remaining 93 basidia, two contained 4 genotypes each for their basidiospores, 17 contained 3 genotypes each, 27 contained 2 genotypes each, and 47 contained only 1 genotype each (Table 1). Genetic linkage analyses of the 23 markers on Chromosome 1 indicated that recombination was observed between all but one adjacent pair of loci (Figure 1) and that the order of the markers on this linkage group matched their relative physical positions on this chromosome. Our markers were placed with approximately 1 marker every 100 kb, yielding an average of 2.9 cM/100 kb.

**Table 1:** Distribution of unique genotypes and inferred haplotypes across the 94 basidia that contained at least one successfully germinated basidiospore.

			Number of basidia with the inferred number of unique haploid genotypes per basidium	
Observed number ofunique genotypesper basidium	Number of basidia with the observed number of genotypes	Inferred number of unique haploid genotypes per basidium	Assuming all progeny are diploid	Assuming hemizygous progeny are aneuploid
1	47	1	9	9
2	26	2	48	48
3	18	3	20	20
4	2	4	8	10
6	1	5	5	5
		6	3	1
		8	1	1

**Figure 2:**
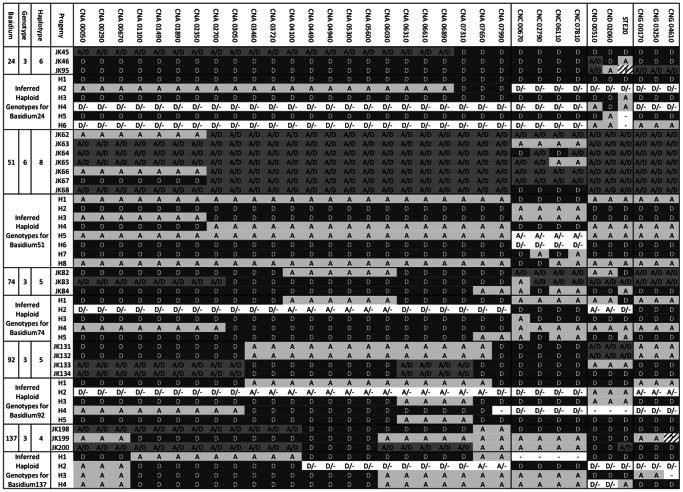
Examples of basidia with progeny genotypes that cannot be explained by the non-disjunction models and the nuclear co-packaging model. Nuclear locus information is presented in the first row and they are arranged sequentially in the same order as that in Table S1 and Figure 1 (for chromosome 1 markers). Allele “A” refers to allele from the serotype A parent; “D” refers to allele from the serotype D parent; “H” refers to a heterozygous locus containing both A and D alleles; “?” Refers to markers where no DNA fragment could be amplified in PCR after three attempts. H1 to H8 are the inferred haploid genotypes for the observed genotypes in the given basidium. When conflict exists between the two approaches for estimating the number of haplotypes (for basidia 17, 92, 137), only the conservative estimate (i.e. one based on the assumption of hemizygous state) is presented. “D/−” and “A/−” in the inferred haploid genotypes refer to two possibilities based on the assumptions used in our inferences: (i) the inferred haplotype has allele A or D if we assume all progeny are diploid; and (ii) the inferred haplotype doesn’t have the locus if we assume those with only one allele are hemizygous, “–” denotes chromosomes/markers which are not present, i.e. a full or partial chromosome loss has occurred. Evidence for mitotic chromosome crossing over is found for chromosome 1 of basidium137, chromosome 3 of basidia 51 and 74, and chromosome 4 of basidium 24.

The observed genotypes were then used to infer the minimum number of haploid genotypes that are needed to explain the observed genotypes for each basidium. Though there are differences in the specific allelic combinations of the inferred genotypes, the two different approaches showed very little difference in the minimum numbers of inferred haploid genotypes for each basidium (Table 1). Of the 94 basidia, three showed differences in the number of haplotypes needed to explain the observed genotypes: basidia17, 92 and 137. For both basidia17 and 92, the PHASE program based on diploid data inferred 6 haplotypes. In contrast, assuming the genotype data as hemizygous, we found that a minimum of 4 and 5 haplotypes respectively could explain basidia17 and 92 (Table S4). For basidium137, the PHASE analysis returned a minimum of five haplotypes while the other analysis resulted in a minimum of four haplotypes. Overall, the hemizygous approach was more conservative (Table 1). Using the conservative hemizygous approach, 87 of the 94 basidia could be explained by having four or fewer haploid genotypes within each basidium. Of the remaining seven basidia, five (basidia 28, 74, 90, 92, and 111) each required a minimum of 5 haploid genotypes to explain their respectively observed genotypes; one (basidium24) required 6 haploid genotypes to explain the three observed genotypes; and one (basidium51) required 8 haploid genotypes to explain the six observed genotypes (Table S4). The observed genotypes and inferred haplotypes for five basidia (basidia24, 51, 74, 92 and 137) are shown in Figure 2. Based on the information in Figure 2 and Table S4, each observed genotype could be reconstituted by a maximum of two inferred haploid genotypes. For example, the genotype of JK45 in basidium 24 can be reconstructed from the haploid genotypes of H1 and H2.

Two processes have been previously found to generate atypical meiotic products in several eukaryotes: Non-disjunction (e.g. 23–25) and meiotic nuclei co-packaging (e.g. 26–28). Below we compare our observed results with the expectations of these two models.

### Comparison of the Genotype Data to the Non-disjunction Models

Non-disjunction is an aberrant meiotic process known to cause abnormal ploidy. It occurs when one (or more) pair(s) of chromosomes fails to segregate during either meiosis I or meiosis II. Non-disjunction has been found in the generation of sexual progeny in a variety of eukaryotes, including many model organisms such as the Zebra fish (*Danio rerio*) [23], maize (*Zea mays*) [24], and the Baker’s yeast (*Saccharomyces cerevisiae*) [25]. While evidence for non-disjunction has not been reported for intra-variety crosses in *C. neoformans*, it has been suggested as the mechanism behind the diploidy/aneuploidy in the meiotic progeny of hybrid crosses between strains of serotypes A and D [15], [22].

Among the 94 basidia containing successfully germinated basidiospores, 47 had only one genotype each and these were not analyzed further for model comparisons because the limited information was insufficient to refute any of the models. The remaining 47 basidia with two or more observed basidiospore genotypes were individually compared to the expectations of the non-disjunction models to determine whether these models could be the sole mechanism(s) to account for the observed genotypes for each basidium. We considered non-disjunction at both meiosis I and meiosis II. Specifically, if non-disjunction occurred during meiosis I, then recombinant non-sister chromatids should be found in the same basidiospore. In addition, each basidium should contain two reciprocal diploid genotypes or reciprocal aneuploidy genotypes for each of the four analyzed chromosomes. Furthermore, the presence of individual basidiospores revealing the same recombination events, but with different degrees of heterozygosity for a given chromosome would be inconsistent with the model. Of the 47 basidia, 17 could be explained by nuclear non-disjunction during meiosis I while 30 could not (Table S3). If non-disjunction occurred during meiosis II, then only sister chromatids would be found together. Our analyses of the observed genotypes for individual basidia showed that 36 of the 47 basidia couldn’t be explained by non-disjunction during meiosis II (Table S3). In total, the genotypes for 30 of the 47 basidia could not be explained by either non-disjunction models (Table S3).

### Comparison of the Genotype Data to the Nuclear Co-Packaging Model

The nuclear co-packing model was proposed to describe sexual reproduction in the commercial button mushroom, *Agaricus bisporus* [26], [27], [28]. This mechanism for generating atypical meiotic progeny refers to the process where the four haploid meiotic daughter nuclei are packaged as pairs into basidiospores, with a bias towards the pairing of non-sister nuclei into the same spore. Sister nuclei refer to the two daughter nuclei formed during the second round of meiosis with both derived from the same nucleus generated after meiosis I. Thus the expectations of this model are that: (i) most basidiospores should each have two nuclei; (ii) the minimum number of inferred haplotypes from each basidia should not exceed four; and (iii) each basidium should have two main highly heterozygous genotypes. However, a recent study found that a high percentage of chromosomes/chromosomal segments in the hybrid progeny of the serotype A and D cross were homozygous [15]. Thus, an alternative nuclear co-packaging scenario is also possible where the daughter nuclei are co-packaged at random. If the co-packaging is random, the four haploid daughter nuclei could be packaged to produce 6 potential diploid genotypes for the basidiospores from a single basidium. Furthermore, each of the four individual haploid nuclei could form a basidiospore and potentially generate a total of 10 different genotypes on a single basidium.

**Figure 3:**
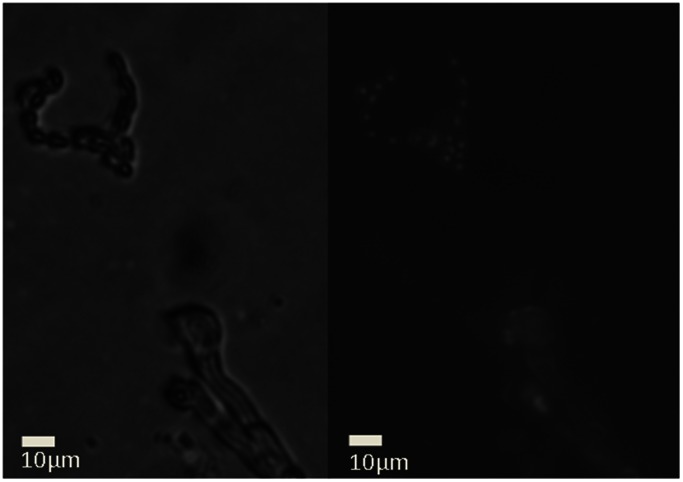
Lack of evidence for binucleated basidiospores in a hybrid cross between strains of serotypes A and D in *C. neoformans*. Left: Standard light image. Right: DAPI and Calcofluor White fluorescence. Scale bar: 10 µm.

We compared the 47 aforementioned basidia that contained two or more observed genotypes each to the expectations of nuclear co-packaging after meiosis. Among the 47 basidia, the co-packaging model (either random or non-random) could explain the genotypes of 40 basidia (Table S3). However, the co-packaging model could not explain the observed genotypes of the remaining seven basidia, due to the greater than four haploid genotypes that were needed to reconstitute the observed genotypes from each of the seven basidia. Furthermore, over 1000 basidiospores were examined under microscope and none were observed to have two nuclei (Figure 3). Of the 230 germinated and analyzed basidiospores, 64 were completely homozygous at all the 33 examined loci (Table S3) while the remaining 166 were heterozygous for at least one of these loci. Assuming that the 64 basidiospore progeny were haploid (and thus uninucleated) and were representative of the overall spore population, we should expect 72% (166/230) of all examined basidiospores to be binucleated based on the co-packaging hypothesis but without nuclear fusion in the basidia). However, 0% was observed to be binucleated. Therefore, to make this model work, we would have to modify it to indicate that the co-packaged nuclei fused immediately after they were co-packaged into basidiospores and before their germination.

In summary, based on the conservative approach, seven basidia each required more than four haploid genotypes to explain the observed genotypes of their basidiospores. Unless we assumed that complete or partial chromosome loss had occurred immediately after germination and that all cells with the ancestral genotype had died out prior to the DNA extraction, none of these seven basidia could be explained through either the non-disjunction or the co-packaging models proposed above. As the cells were only subcultured once after germination, we believe this scenario is extremely unlikely. Instead, we believe other mechanism(s) were likely involved to cause the observed genotypes and these mechanisms are discussed below.

### Potential Mechanisms

In the first potential model, there might be more than one round of meiosis in each basidium in our hybrid cross. However, a previous study by Idnurm *et al.* (2010) [9] has shown that in intra-variety crosses between strains of serotype A in *C. neoformans*, a maximum of four haploid genotypes were found for basidiospores from each basidium and thus only one round of meiosis was needed in each basidium to generate their genotypes. Indeed, as far as we know, there has been no report for more than one round of meiosis for any taxa in the generation of an individual gamete. While possible, this hypothesis would require an additional new regulatory pathway to initiate a second round of meiosis soon after the first one ended in the basidia. In addition, without other processes, the additional meiosis by itself could not generate the prevalent heterozygous genotypes observed here. Thus, we believe this model is extremely unlikely.

**Figure 4:**
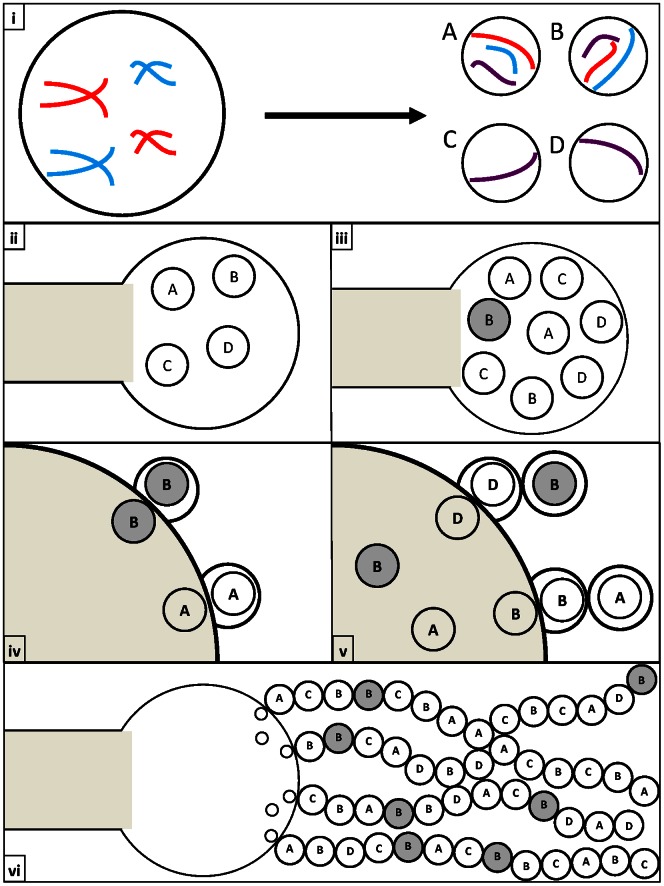
Non-disjunction followed by mitotic recombination within the basidium. (i) Mis-segregation occurs during meiosis to produce some nuclei with additional chromosome copies (n+x) and some without the corresponding chromosomes (n−x). [Purple chromosomes represent copies which have undergone recombination]. (ii) & (iii) The four diploid/aneuploid daughter nuclei produce additional copies of themselves through mitosis. At this point some nuclei go through mitotic recombination (Grey circles) to form novel genotypes. (iv) & (v) The daughter nuclei migrate to the edge of the basidium where they go through an additional round of mitosis to produce a nucleus which is packaged into a basidiospore. (vi) Nuclei divide and stochastically migrate towards the basidiospores forming long heterogeneous chains of diploid/aneuploid basidiospores.

The second possibility involves both non-disjunction during meiosis I and mitotic recombination within nuclei after meiosis II but before the nuclei enter into basidiospores. In this model, there is non-disjunction during meiosis I in these basidia that would generate two diploid heterozygous nuclei at the end of meiosis II. During subsequent mitotic divisions to generate progeny nuclei for basidiospores, recombination could occur between non-sister homologous chromosomes which could produce a diversity of diploid/aneuploid genotypes for basidiospores (Figure 4).

The third possibility involves non-disjunction during meiosis II, and followed by nuclei fusion and mitotic recombination within the basidia. In this model, the non-disjunction during meiosis II would generate haploid nuclei with additional or missing chromosomes. These nuclei would then fuse within the basidia and during subsequent mitotic divisions to generate progeny nuclei for basidiospores, recombination could occur between non-sister homologous chromosomes which could produce a diversity of diploid/aneuploid genotypes for basidiospores.

**Figure 5:**
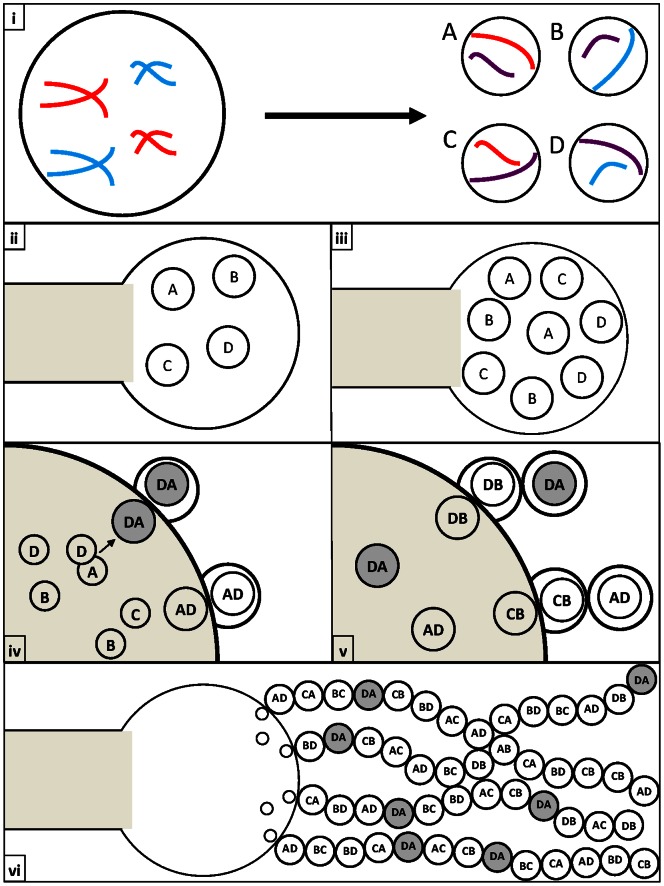
Non-random co-packaging and fusion of daughter nuclei followed by mitotic recombination within the basidium. (i) Meiosis occurs properly to form four haploid daughter nuclei. [Purple chromosomes represent copies which have undergone recombination]. (ii) & (iii) The four haploid daughter nuclei produce additional copies of themselves through mitosis. (iv) & (v) The daughter nuclei fuse to form diploid nuclei. Subsequent rounds of mitosis produce multiple copies of the diploid nuclei. Through these mitotic divisions, recombinant genotypes (Grey circles) may be produced and packaged into basidiospores. (vi) Nuclei divide and stochastically migrate towards the basidiospores forming long heterogeneous chains of diploid/aneuploid basidiospores.

The fourth possibility involves a regular meiosis to generate four haploid nuclei within each basidium but followed by preferential fusion of non-sister haploid nuclei into two diploid nuclei, and then through mitosis to generate multiple diploid nuclei to enter into basidiospores. During replication of the diploid nuclei within the basidium, mitotic recombination could be involved to produce a diversity of genotypes, consequently leading to greater than four inferred haploid nuclear genotypes (Figure 5).

Our current genotype data are unable to distinguish the proposed models 2, 3 and 4. Instead, we believe detailed cytological observations of nuclear and chromosomal dynamics within basidia are needed in order to understand the underlying processes involved in generating the diversity of genotypes. However, potential models 2, 3, and 4 all require mitotic recombination within the basidia, before the nuclei enter into basidiospores. Indeed, the proposed mitotic recombination could generate a large number of unique genotypes within a given basidium, regardless of their original ploidy and/or genotypic state after meiosis. If true, the result would suggest that the rate of meiotic recombination would likely be lower than what was determined by Sun *et al.* (2007) [15], which already showed a decreased in recombination as compared to the intra-variety cross.

### Mitotic Recombination within Basidia Likely Includes both Chromosomal Loss and Crossing Over

Based on genotype comparisons, the inferred mitotic recombination within basidia likely included both chromosomal loss and crossing over. Differences in the patterns of heterozygosity among the chromosomes within many of the individual basidiospores are consistent with chromosomal losses (Tables S3 and S4). However, to identify mitotic chromosome crossing over within individual basidia, we looked for basidium that contained more than four reciprocal haploid genotypes for an individual chromosome. Four reciprocal haploid genotypes per chromosome are the maximum of what we should expect from a single round of meiosis and deviations from this expectation would be consistent with mitotic chromosomal crossing over within the basidium.

**Figure 6:**
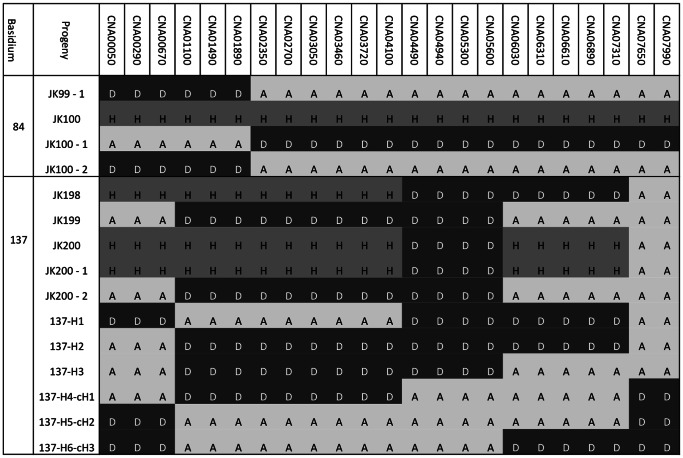
Evidence for reciprocity of recombinant haploid genotypes (basidium 84) and for mitotic chromosomal crossing-over within Chromosome 1 (basidium 137). JK100-1 and JK100-2 were two subcultures from a single basidiospore (JK100) of basidium 84. Basidiospore JK100 was completely heterozygous for all the marker loci on chromosome 1. However, analyses of the two subcultures identified that the two subcultures showed reciprocal genotypes for chromosome 1 with JK100-2 showing an identical genotype to the genotype of a different spore JK99. JK200-1 and JK200-2 were two subcultures from a single basidiospore JK200. Four recombination breakpoints were identified for chromosome 1 within this basidium and a minimum of three Chromosome 1 haploid genotypes (137-H1 to 137-H3) was inferred for this basidium to explain the observed genotypes. However, meiosis generates reciprocal recombinant genotypes. Thus, three additional Chromosome 1 genotypes are further inferred. Haploid genotypes 137-H4-cH1, 137-H5-cH2, and 137-H6-cH3 are the reciprocal genotypes for 137-H1, 137-H2, and 137-H3 genotypes respectively. Since each round of meiosis generates a maximum of four haploid nuclear genotypes, mitotic chromosomal crossing-over must be involved to produce the six haploid genotypes chromosome 1.

Our screening revealed that four basidia (24, 51, 74, and 137, Figure 2) each had one chromosome that contained more than four reciprocal haploid nuclear genotypes (inferred haploid genotype plus the reciprocals of the inferred haploid genotypes if the reciprocals are not present in the inferred genotype group already). Specifically, Chromosome 4 (represented by three CND markers) in basidium24 contained five inferred haploid genotypes plus five reciprocals of the inferred five; Chromosome 3 (represented by four CNC markers) in basidium51 contained four inferred plus 2 additional reciprocals; Chromosome 3 in basidium74 contained four inferred plus 2 additional reciprocals; and Chromosome 1 in basidium137 contained three inferred and three additional reciprocals) (Figure 2 and Figure 6).

An example of reciprocal chromosome genotype inference is shown in Figure 6 for basidium137. In this basidium, basidiospore JK200 was subcultured and two segregant colonies were genotyped for Chromosome 1. Subculture JK200-1 was found to have an identical genotype to JK200 while JK200-2 showed the loss of heterozygosity on chromosome 1 (Figure 6; Table S4). This loss of heterozygosity allowed us to infer the exact allelic composition of the two copies of Chromosome 1 within the original basidiospore JK200 as well as the minimum number of haploid genotypes that are required to reconstitute the observed genotypes for basidium137. Our analyses identified a total of four recombination breakpoints and a minimum of three haploid genotypes for chromosome 1 within basidium137 (Figure 6). However, none of these three haploid genotypes represented reciprocal genotypes of each other (as would be expected from meiosis) and three other reciprocal haploid genotypes were thus required to fully reconstitute the meiotic process. These observations are thus consistent with mitotic chromosome crossing-over within this basidium that contributed to generating the six haploid genotypes for chromosome 1.

Direct evidence for reciprocity of recombinant genotypes was found in a progeny from basidium84 that originally typed ambiguously, i.e. multiple markers on chromosome 1 showed heterozygosity but with one of the two alleles (DNA fragments on the gel) being weaker than the other allele at all the marker loci on this chromosome. The progeny (JK100) was subcultured and the obtained pure subcultures were re-typed for markers on chromosome 1, which resulted in two genotypes (Figure 6). Specifically, JK100-1 and JK100-2 were purified from the same colony formed by a single basidiospore and they were found to possess reciprocal genotypes at chromosome 1. Interestingly, subculture JK100-2 had the same genotype at chromosome 1 as an independent progeny JK99-1 from the same basidium, which strongly suggests that the reciprocity was generated during meiosis and that chromosome 1 of JK100-1 and JK100-2 were likely co-packaged together into the same basidiospore JK100. Alternatively, mitotic crossing over after germination could also give rise to the reciprocal genotypes. However, this scenario is very unlikely and would require that the meiotic recombination (as shown in basidiospore JK99) within the basidium and the mitotic recombination after germination (for basidiospore JK100) to happen within the same chromosomal region between adjacent markers CNA01890 and CNA02350.

We would like to note that for several reasons, the observed and inferred numbers of genotypes shown here were likely underestimates of the true genotype diversities and recombination processes. First, only four of the 14 chromosomes were genotyped and with the majority of the markers on one chromosome. If more markers were genotyped on these four chromosomes as well as with markers on the remaining 10 chromosomes, more genotypes would likely be found for spores from each basidium. Second, basidiospores that did not germinate likely contained other genotypes that we had missed from all basidia. Third, at present, the inferred haploid genotypes for each basidium were the minimum set required to re-constitute the observed genotypes. A greater number of haplotypes could have existed for these and other spores from each basidium. More detailed analyses using more markers will likely increase the number of observed and inferred genotypes substantially and lead to greater evidence for mitotic recombination within these basidia.

Since early in their discovery, it has been suspected that the genomes of the AD hybrids could undergo mitotic recombination to generate novel genotypes, as serotyping of isolates after subsequent sub-culturing can lead to different results [29]. If chromosome loss occurs through mitotic recombination within the basidium, it is possible that the same mechanisms could revert AD hybrids to serotype A or D during sporulation. This would imply that the genomes of AD hybrids are inherently flexible as opposed to varying solely in response to stress and selective pressure. In addition, mitotic recombination within basidia could potentially subvert any mechanisms which may have evolved to suppress recombination during meiosis between the two varieties, preventing the varieties from undergoing full speciation within sympatric populations and increasing the viability and genetic diversity of recombinant hybrids.

## Supporting Information

Table S1 **Information for the 33 genetic loci analyzed in this study.** Column A: marker code (1–33); column B: locus code/name as annotated in the *C. neoformans* var. *neoformans* (serotype D) strain JEC21 genome; column C: primer sequences; columns D: locus location on the annotated *C. neoformans* var. *grubii* (serotype A) strain H99 genome; column E: size of amplified fragment for the parent strain CDC15; columns F: locus location on the annotated *C. neoformans* var. *neoformans* (serotype D) strain JEC21 genome; column G: size of amplified fragment for the parent strain JEC20; column H: restriction enzyme used to distinguish the parental alleles.
(XLS)

Table S2 **Summary information for the numbers of basidiospores dissected and germinated from the 194 basidia.**
(XLS)

Table S3 **Genotypes of all 230 germinated basidiospores.** The genotype data are organized based on their basidium affiliation. Also included in the table are the number of genotypes observed for spores from each basidium (column B), the number of inferred haplotypes based on the observed genotypes of basidiospores (column C), and whether the known meiotic mechanisms could explain the observed and inferred genotypes (Columns D, E, and F). Y: the known mechanism can explain the observed results; N: the specific mechanism cannot explain the observed results. The details of the three mechanisms are discussed in the Main Text.
(XLS)

Table S4 **The observed genotypes and inferred haplotypes for spores from nine basidia that suggested evidence for mitotic recombination within basidia.**
(XLS)
